# Association between cigarette smoking and the risk of dysmenorrhea: A meta-analysis of observational studies

**DOI:** 10.1371/journal.pone.0231201

**Published:** 2020-04-15

**Authors:** Lu-Lu Qin, Zhao Hu, Atipatsa Chiwanda Kaminga, Bang-An Luo, Hui-Lan Xu, Xiang-Lin Feng, Jia-He Liu

**Affiliations:** 1 Department of Social Medicine and Health Management, School of Medicine, Hunan Normal University, Changsha, China; 2 Department of Social Medicine and Health Management, Xiangya School of Public Health, Central South University, Changsha, China; 3 Department of Mathematics and Statistics, Mzuzu University, Luwinga, Mzuzu, Malawi; 4 Department of Epidemiology and Health Statistics, Xiangya School of Public Health, Central South University, Changsha, Hunan, China; 5 Department of Mental Health, Brain Hospital of Hunan Province, Changsha, Hunan, China; University of Calfornia San Francisco, UNITED STATES

## Abstract

**Background:**

Emerging studies have found inconsistent results on the potential relationship between cigarette smoking and dysmenorrhea. Therefore, the aim of this study was to quantitatively synthesize the previous findings on the preceding relationship using meta-analysis.

**Methods:**

Previous studies on the association between cigarette smoking and dysmenorrhea, published not later than November 2019, were systematically searched, using MeSH heading and/or relevant terms, in the electronic databases of PubMed, Medline, Web of Science and EMBASE. The *I*^*2*^ statistic was used to assess heterogeneity, whose source was explored using subgroup analysis. A pooled effect size was obtained using random effects model, and sensitivity analysis was performed to assess the consistency of the pooled effect size.

**Results:**

After a rigorous screening process, 24 studies involving 27,091 participants were included in this meta-analysis. The results indicated that smokers were 1.45 times more likely to develop dysmenorrhea than non-smokers (odds ratio (OR) = 1.45, 95% confidence interval (CI): 1.30–1.61). In addition, individuals classified as currently smoking were 1.50 times more likely to develop dysmenorrhea than those who were classified as never smoking (OR = 1.50, 95% CI: 1.33–1.70), whereas being a former smoker was 1.31 times more likely to develop dysmenorrhea than being a never smoker (OR = 1.31, 95% CI: 1.18–1.46). Sensitivity analysis showed that exclusion of any single study did not materially alter the overall combined effect.

**Conclusion:**

The evidence from this meta-analysis indicated a significant association between cigarette smoking (both current and former smoking) and dysmenorrhea. The adverse effects of smoking provide further support for prevention of dysmenorrhea and emphasize the need to target women.

## 1. Introduction

Dysmenorrhea is the most common gynaecological disorder worldwide, with a prevalence ranging from 50% to 90% according to different countries [1–4]. The pelvic or lower abdominal pain is one of the most common symptoms of dysmenorrhea, which usually lasts for three days from the beginning of menstruation. Dysmenorrhea is classified as primary or secondary [5]. Primary dysmenorrhea (PD) refers to painful menses or cramps in the lower abdomen before and/or during menstruation without an identifiable organic pathology [6]. In contrast, secondary dysmenorrhea refers to menstrual pain resulting from anatomic and/or evident pelvic pathology, such as endometriosis [7]. The discomfort brought about by dymenorrhea has negatively affected women’s quality of life and their performance in everyday activities. In addition, the prevalence of dysmenorrhea causes heavy medical burden, such as serious health costs [8]. Therefore, dysmenorrhea is an urgent public health problem. Nevertheless, about more than 10% of adolescent girls have severe dysmenorrhea, which is the main reason for school and work absenteeism among the adolescent girls [9]. However, only a few women seek medical help for this disorder.

Despite the preceding evidence on the prevalence of dysmenorrhea, factors influencing dysmenorrhea over the reproductive life span are not fully understood. In spite of that, hitherto the condition of dysmenorrhea correlates with a number of factors, such as family history of dysmenorrhea, age of menarche onset, caffeine consumption, dietary habits, exercise, cigarette smoking, and some psychological or gynaecological factors [10].

Cigarette smoking has been reported in literature as one of the modifiable risk factors for dysmenorrhea. Although women who smoke reported a range of more adverse reproductive outcomes than their non-smoking counterparts, the relationship between smoking and dysmenorrhea is still heterogeneous [11, 12]. That is, some studies found that smokers were more likely to experience dysmenorrhea than non-smokers, whereas other studies reported that smokers were less likely to experience dysmenorrhea than non-smokers, and another study showed that smoking had no effect on dysmenorrhea. For instance, a study conducted in Turkey reported that smokers had a 1.6-fold higher risk of dysmenorrhea than non-smokers [13]. This was consistent with an earlier study, which found that women smokers experienced more severe dysmenorrhea, whose degree varied with the number of cigarettes smoked every day [14]. Furthermore, Parazzini et al. reported that the risk of dysmenorrhea increased with the duration of smoking and the number of cigarettes smoked every day [15]. In contrast, Andersch et al. reported that smokers had significantly less dysmenorrhea than non-smokers [16]. In addition, other three studies (two conducted in Turkey and one conducted in Japan) did not find an association between smoking and dysmenorrhea among women [17–19].

In this regard, the aim of this study was to quantitatively synthesize the previous findings on the association between cigarette smoking and dysmenorrhea and give further suggestions on interventions for dysmenorrhea.

## 2. Methods

### 2.1 Search strategy

This meta-analysis was conducted in accordance with the guidelines for observation study protocols (Meta-analysis Of Observational Studies in Epidemiology (MOOSE) guidelines). A systematic search for literature was conducted in the electronic databases of PubMed, Medline, Web of Science, and EMBASE to locate related studies, published not later than November 2019, using the following keywords: “smoking” or “cigarette smoking” or “tobacco” or “nicotine” or “smoke” or “risk factors” in combination with “dysmenorrhea” or “menstruation pain” or “menstruation disorder” or “painful menstruation” or “menstrual painful” or “menstrual dysfunction”. Besides, reference lists of eligible studies were searched manually for relevant studies.

### 2.2 Study selection

Studies were considered eligible for inclusion in this meta-analysis if they fulfilled the following inclusion criteria: (a) observational studies published in English, which evaluated the main outcome as the relationship between cigarette smoking and dysmenorrhea, or the influencing factors of dysmenorrhea including cigarette smoking; (b) studies assessed the existence of dysmenorrhea in the smoking and non-smoking groups; and (c) studies presented the sample sizes, number of cases, and odds ratios (ORs) with 95% confidence intervals (CIs). The exclusion criteria of studies were as follows: (a) reviews, studies not on humans, abstracts, and comments; (b) studies on subjects experiencing passive smoking; (c) studies with inadequate information for this meta-analysis; and (d) duplicates of already included studies.

### 2.3 Data extraction

Two authors (Lu-Lu-Qin, Zhao Hu) independently selected eligible studies by screening the titles, abstracts and full texts according to the eligibility criteria. Any discrepancies on the selected studies were resolved by consensus with another author (Bang-An Luo), who reviewed again the different selections and made decisions or agreements based on the eligibility criteria. Then, the following data were extracted from all the studies which met the eligibility criteria: (a) name of first author; (b) publication year; (c) location of the study; (d) sample size; (e) study design; (f) age; (g) type of dysmenorrhea; (h) prevalence of dysmenorrhea; (i) definition of exposure; and (j) outcome. The main variables for assessing the relationship between cigarette smoking and dysmenorrhea were as follows: (a) number of smokers (current and ever); (b) number of females who smoked cigarettes and had dysmenorrhea; (c) number of non-smokers; and (d) number of females who did not smoke and had dysmenorrhea. Extracted data were recorded in standardized tables and confirmed by two investigators separately (Lulu Qin, Zhao Hu).

### 2.4 Quality assessment

The methodological quality of cross-sectional studies was assessed using the tool, the Agency for Healthcare Research and Quality (AHRQ). Studies assessed using this tool are classified as high quality (if they scored 8–11), moderate quality (if they scored 4–7) or low quality (if they scored 0–3). The methodological quality of cohort or case-control studies was assessed using the New-Ottawa Scale (NOS). Studies assessed using the NOS are classified as high quality (if they scored 7–9), moderate quality (if they scored 4–6) or low quality (if they scored 0–3).

### 2.5 Statistical analysis

The ORs together with their corresponding 95% CIs were calculated using the Mantel-Haenszel procedure for the comparisons of dichotomous outcomes between the smoking and non-smoking groups. The heterogeneity among the results of the included studies was evaluated using the *I*^2^ statistic tests. A random-effects model was chosen to calculate the pooled ORs, when there was substantially moderate or high heterogeneity among the included studies. Besides, sensitivity analysis was performed by excluding one study at a time in order to assess the consistency of the pooled estimate of the ORs. Also, the possibility of publication bias was assessed using a funnel plot and the Begg’s and Egger’s tests (significance level, *p*<0.05). Furthermore, subgroup analysis was conducted to explore potential heterogeneity according to study design, study location, type of dysmenorrhea and study quality. Only two studies reported the number of cigarettes smoked; thus, we did not analyse the dose-response association between the degree of smoking exposure and dysmenorrhea due to insufficient data. Most statistical analyses were performed in RevMan Software (Version 5.3, Cochrane Collaboration, London, UK). The publication bias test and subgroup analysis were conducted in STATA version 12.0 (StataCorp, College Station, TX).

## 3. Results

### 3.1 Study selection

**Fig 1:**
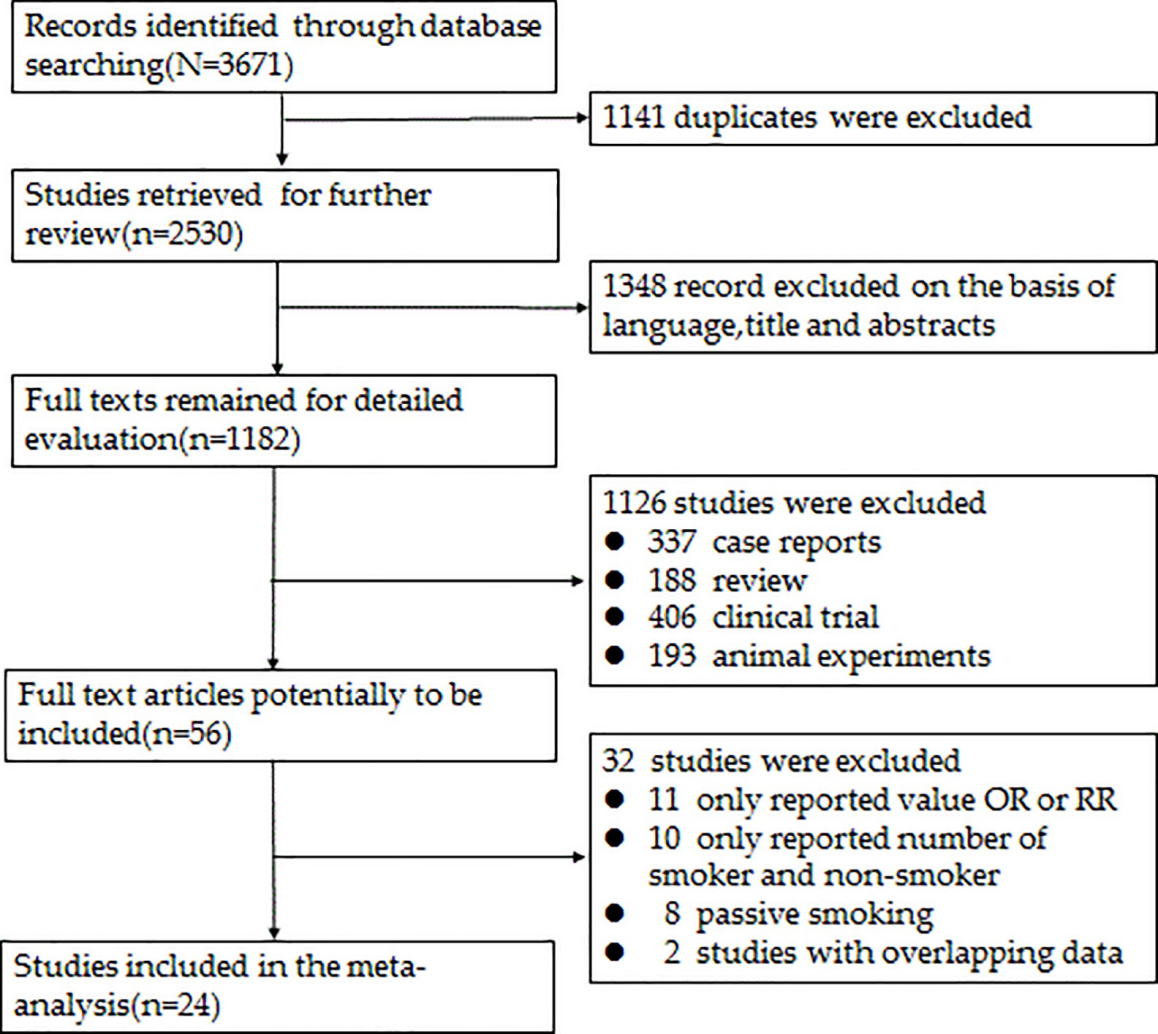
Flow chart of literature search and study selection.

The search strategy yielded a total of 3,671 studies, of which 24 were selected as eligible for this meta-analysis following a rigorous screening process (Fig 1).

### 3.2 Study characteristics

Characteristics of the 24 eligible studies are displayed in Table 1. Among these studies, the diversity of participants’ characteristics was considerable. Altogether, these studies investigated 27,091 female participants, among whom 11,731 (43.3%) had dysmenorrhea. In addition, among the 24 eligible studies, there were 4 case-control studies, 2 cohort studies and 18 cross-sectional studies. Moreover, 10 studies were conducted in Europe, 9 in Asia, 3 in North America and 2 in Oceania. The sample sizes of the eligible studies ranged from 258 to 9,067; while age of the participants varied between 15 and 59 years. Considering the individual eligible studies, the prevalence of dysmenorrhea varied between 20.1% and 91.8%. Also, 8 studies investigated cigarette smoking and the risk of primary dysmenorrhea, whereas others explored cigarette smoking and the risk of nonspecific dysmenorrhea. Many of these studies defined smokers as smoking at least one cigarette per day or they described the current smoking status of the participants, while few did not provide a clear definition. Furthermore, some studies defined dysmenorrhea as cramps or abdominal pain or backache one day before and/or the first day of menstruation, while other studies considered dysmenorrhea to be an activity-limiting pain that requires medication. Almost all studies used a self-administered questionnaire to measure the outcomes of dysmenorrhea with respect to smoking status. Additionally, 2 case-control studies and 2 prospective cohort studies were of high quality, whereas other 2 case-control studies were of medium quality according to the assessment tool of NOS (S1 and S2 Tables). Also, among the 18 cross-sectional studies, 5 studies were of high quality, 10 studies were of medium quality, and 3 studies were of low quality, following the evaluation with the AHRQ tool (S3 Table).

**Table 1:** Characteristics of the eligible studies for this meta-analysis.

Author and year	Location of study	Study design	Sample size	Age(years)*	Type of dysmenorrhea	Prevalence of dysmenorrhea (%)	Definition of smoking	Definition of dysmenorrhea	Methodological quality(score)
Wood C(1979)[20]	Australia	Cross-sectional	699	15–59	Not specified	45.7	Never/former/current smoker	Any pain	Low(3)
Teperi J(1989)[21]	Finland	Cross-sectional	3370	12–19	Not specified	75.5	No/occasionally/daily smoking	Any pain	Medium(4)
Sundell G(1990)[14]	Sweden	Cross-sectional	489	19–24	Primary	67.0	NA	Cramps	High(8)
Parazzini F(1994)[15]	Italy	Case-control	251	15–44	Primary	41.4	NA	Pelvic complaints	High(7)
Charlton A(1996)[22]	England	Cross-sectional	2181	16–17	Not specified	56.4	Never/sometime/regular smoking	Any pain	Medium(4)
Kritz-Silverstein D(1999)[23]	USA	Cross-sectional	2912	18–49	Not specified	25.1	Smoked at least 1 cigarettes per day	Cramps	High(9)
Strinić T(2003)[24]	Croatia	Cross-sectional	297	11–18	Primary	55.2	Current smoker	Any pain	Medium(4)
Weissman(2004)[25]	USA	Prospective	404	19–46	Primary	76.0	Current smoker	Menstrual cramps	High(7)
Burnett MA(2005)[26]	Canada	Cross-sectional	1546	≥18	Primary	65.7	NA	Any pain	Low(3)
Patel V(2006)[27]	India	Cross-sectional	2262	18–45	Not specified	54.7	NA	Cramps/abdominal/backache pain†	Medium(7)
László KD(2009)[28]	Hungary	Cross-sectional	821	37.2±9.4	Not specified	20.1	Never/former/current smoker	Limiting activity	Medium(6)
Ozerdogan N(2009)[13]	Turkey	Cross-sectional	857	17–32	Not specified	55.5	Smoked at least 1 cigarettes per day	Cramps/abdominal /backache pain†	High(8)
Unsal A(2010)[18]	Turkey	Cross-sectional	623	17–30	Not specified	72.7	Smoked at least 1 cigarettes per day	Abdominal/groin/ lumbar pain	Medium(5)
Wong LP(2010)	Malaysia	Cross-sectional	1092	15.2±1.4	Not specified	74.5	NA	Any pain	Medium(5)
Grandi G(2012)[29]	Italy	Cross-sectional	408	22.9±3.0	Not specified	84.1	NA	Any pain	Medium(5)
Gagua T(2012)[12]	Georgia	Case-control	431	16.0±1.4	Primary	52.1	NA	Any pain	Medium(5)
Ju H(2014)[30]	Australia	Prospective	9067	22–27	Not specified	25.0	Never/former/current smoker	Any pain	High(7)
Sahin S(2014)[31]	Turkey	Cross-sectional	520	17–25	Not specified	69.0	Smoked at least 1 cigarettes per day	Abdomen/thighs /lower back pain	High(8)
Ibrahim NK(2015)[32]	Saudi Arabia	Cross-sectional	435	21.4±1.4	Not specified	60.9	NA	two or more days of menstrual pain during bleeding	Medium(4)
Pejčić A(2016)[33]	Serbia	Case-control	288	18–29	Not specified	84.4	Smoked at least 1 cigarettes per day	Limiting activity/require medication	Medium(5)
Tomás-Rodríguez MI(2017)[34]	Spain	Cross-sectional	306	18–30	Primary	91.8	personal history of smoking	Limiting activity	Medium(4)
Abu Helwa HA(2018)[35]	Palestine	Cross-sectional	956	19.7±1.5	Not specified	85.1	Current smoker	Suprapubic/flank/back/thigh pain	High(8)
Fernández-Martínez E(2018)[11]	Spain	Cross-sectional	258	18–45	Not specified	74.8	NA	Any pain	Low(3)
Orhan C(2018)[36]	Turkey	Case-control	471	19–22	Primary	87.3	Smoking habit	painful menstruation in the last 3 months	High(7)

*data are presented as range or mean±SD; NA: not available

### 3.3 Smoking and risk of dysmenorrhea

**Fig 2:**
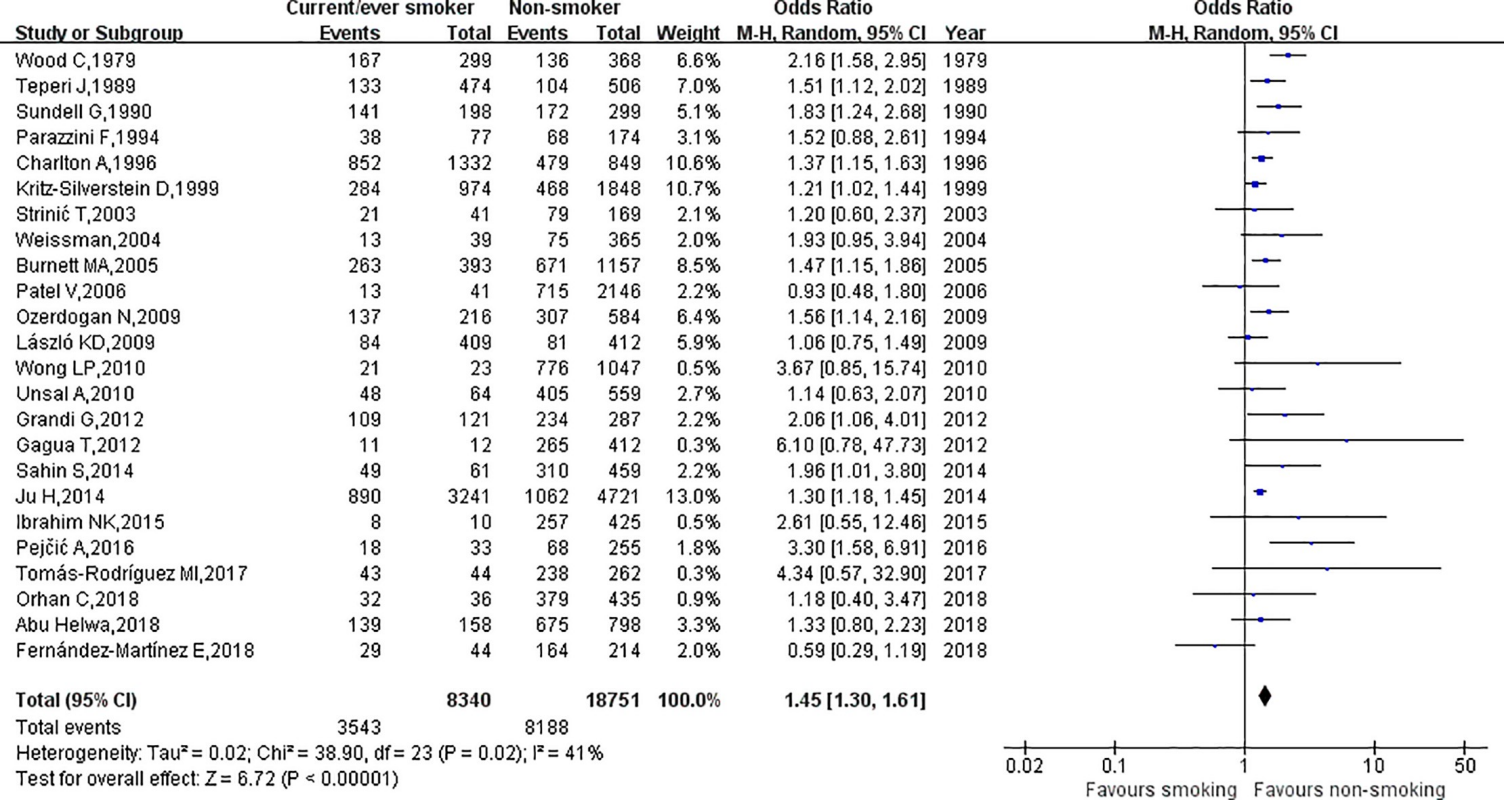
The association between smoking and dysmenorrhea.

**Fig 3:**
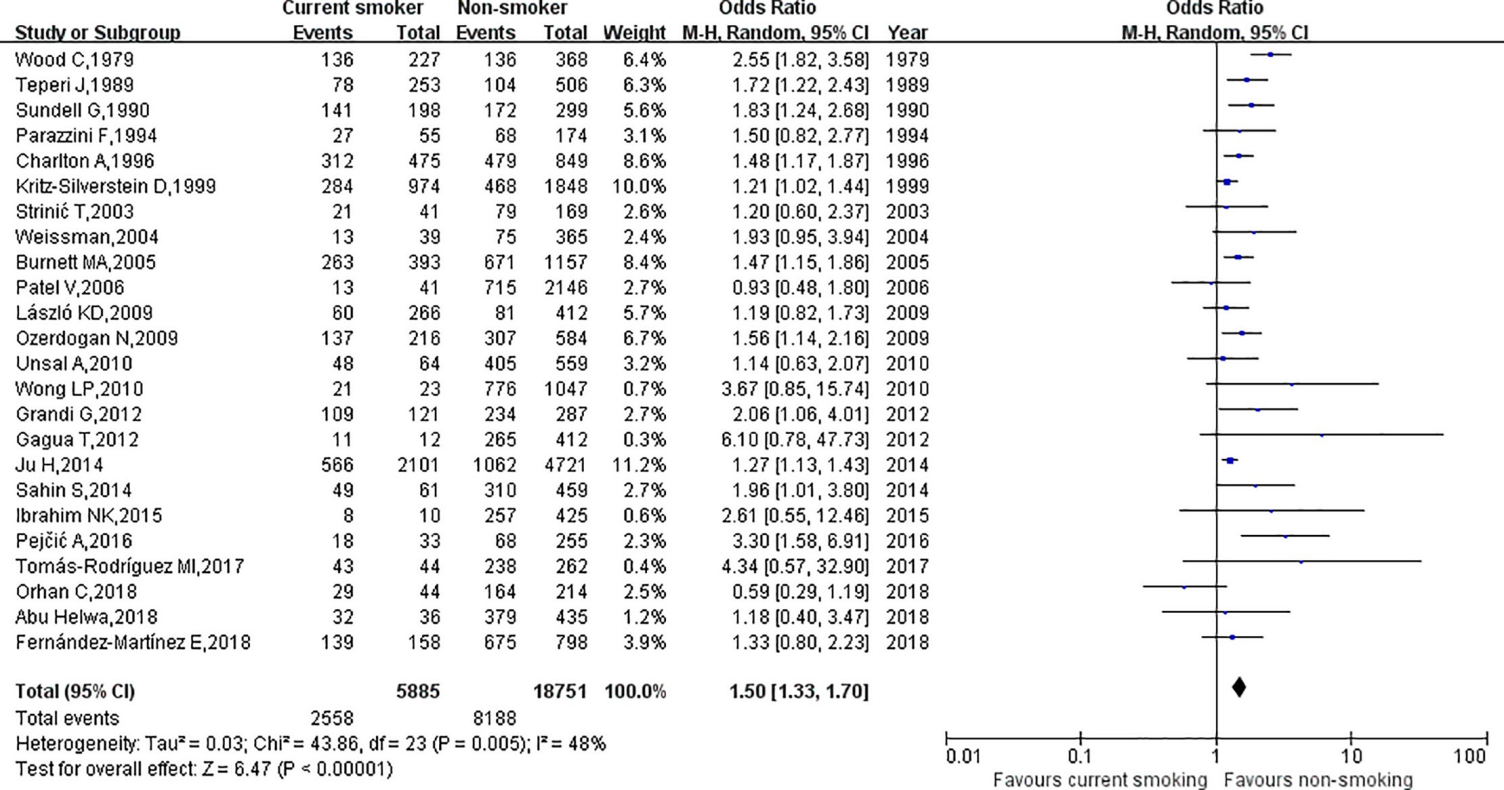
The association between current smoking and dysmenorrhea.

**Fig 4:**
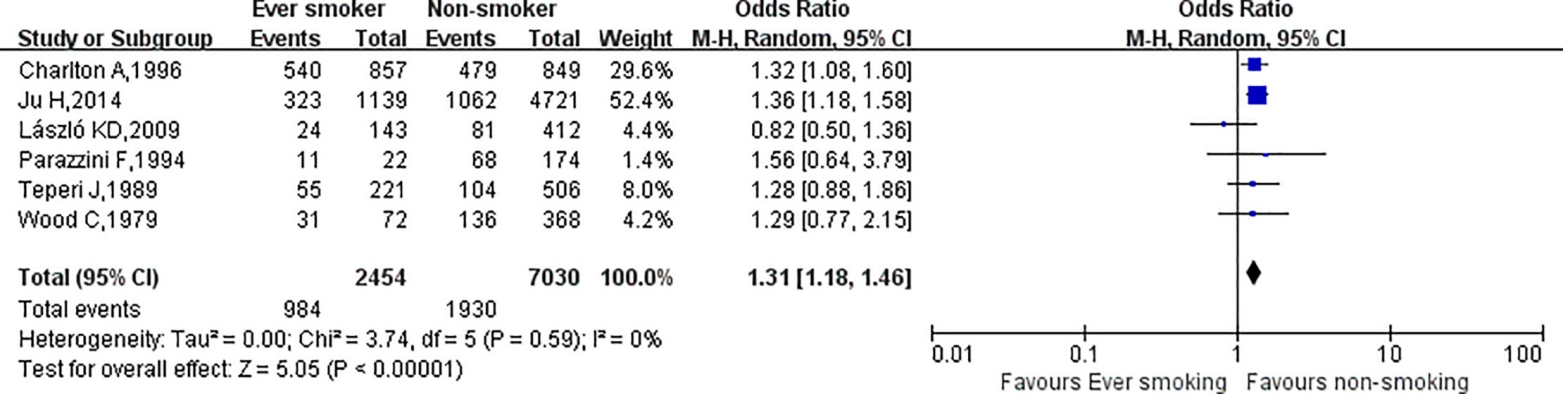
The association between former smoking and dysmenorrhea.

The relationship between cigarette smoking and dysmenorrhea among females is shown in Fig 2. Using random-effects model, the results of this meta-analysis showed that, smokers were 1.45 times more likely to develop dysmenorrhea than non-smokers (OR = 1.45, 95% CI: 1.30–1.61). This result was associated with medium heterogeneity (*I*^2^ = 41.0%, *p* = 0.02). Moreover, individuals classified as currently smoking were 1.50 times more likely to develop dysmenorrhea than those who were classified as never smoking (OR = 1.50, 95% CI: 1.33–1.70) and, again, medium heterogeneity was associated with this result (*I*^2^ = 48.0%, *p* = 0.005). Also, being a former smoker was 1.31 times more likely to develop dysmenorrhea than being a never smoker (OR = 1.31, 95% CI: 1.18–1.46), and no heterogeneity was associated with this result (*I*^2^ = 0%, *p* = 0.59). The results are shown in Fig 3 and Fig 4.

### 3.4 Subgroup and sensitivity analyses

The results of sensitivity analysis indicated that inclusion and exclusion of Wood`s study [20] significantly affected the magnitude of the heterogeneity associated with the pooled OR. That is, after excluding this study, the pooled OR was reduced but still significant (OR = 1.39, 95% CI: 1.26–1.54), and with reduced heterogeneity (*I*^*2*^ = 28%). Besides, the pooled results were still significant after excluding one study at a time.

Furthermore, results of subgroup analyses are shown in Table 2. As regards prospective cohort studies, the pooled OR (OR = 1.35, 95% CI: 1. 09–1.67) was lower than that of case-control studies (OR = 2.03, 95% CI: 1.15–3.57) or cross-sectional studies (OR = 1.43, 95% CI: 1.26–1.62) (S1 Fig). Comparable pooled ORs were observed among studies conducted in Europe (OR = 1.45, 95% CI: 1.17–1.78), Asia (OR = 1.47, 95% CI: 1.19–1.81), North America (OR = 1.34, 95% CI: 1.12–1.61) and Oceania (OR = 1.64, 1.00–2.68) (S2 Fig)

**Table 2:** Pooled ORs and heterogeneity in subgroup analyses.

Variables	No.studies	OR(95% CI)	*I*^2^%	P for heteogeneity
Study design				
Cross-sectional	18	1.43 (1.26,1.62)	41.8	0.033
Case-control	4	2.03 (1.15,3.57)	37.3	0.188
Cohort	2	1.35 (1.09,1.67)	13.4	0.283
Location of study				
Asia	9	1.47 (1.19,1.81)	0.0	0.470
Europe	10	1.45 (1.17,1.78)	51.5	0.029
North American	3	1.34 (1.12,1.61)	26.6	0.256
Oceania	2	1.64 (1.00,2.68)	89.0	0.003
Type of dysmenorrhea				
Primary	8	1.56 (1.31,1.86)	0.0	0.686
Not specific	16	1.41 (1.24,1.61)	52.8	0.007
Study methodological quality				
High	9	1.34 (1.23,1.45)	0.0	0.485
Medium	12	1.45 (1.19,1.77)	35.2	0.109
Low	3	1.36 (0.81,2.28)	83.2	0.003

Considering type of dysmenorrhea, smoking increased the risk of primary dysmenorrhea by 56% among the females (OR = 1.56, 95% CI: 1.31–1.86) (S3 Fig). The pooled OR of studies with high methodological quality (OR = 1.34, 95% CI: 1.23–1.45) was lower than that of studies with medium methodological quality (OR = 1.45, 95% CI: 1.19–1.77) and those with low methodological quality (OR = 1.36, 95% CI: 0.81–2.28) (S4 Fig).

### 3.5 Publication bias

According to the results of the Begg’s test (*z* = 1.66; *p* = 0.097) and Egger’s test (*t* = 1.97, *p* = 0.061), there was no significant publication bias. However, the fact that the preceding results were closer to being significant, there might be moderate or non-obvious publication bias as shown by the funnel plot (S5 and S6 Figs).

## 4. Discussion

According to the results of this meta-analysis, there is strong evidence that cigarette smoking is significantly related to an increased risk of dysmenorrhea among women of reproductive age (OR = 1.45, 95% CI: 1.30–1.61). This precise estimation of smoking as a risk factor for dysmenorrhea offers a new approach for prevention of dysmenorrhea among women of reproductive age. There are two previous meta-analyses on the association between cigarette smoking and dysmenorrhea [34, 35]. However, to the best of our knowledge, this is the first meta-analysis to synthesize evidence on the relationship between smoking (including current and former smoking) and dysmenorrhea, using the most comprehensive available evidence in literature. For example, unlike in this study, in the study conducted by Latthe et al [34], the number of included studies was limited, and the publication bias was clear. Besides, heterogeneity, subgroup and sensitivity analyses were not reported. Also, in the study by Jenabi et al [37], only the relationship between current smoking and dysmenorrhea among females was studied, for which subgroup and sensitivity analyses were lacking to explore potential heterogeneity. Thus, this meta-analysis represents the best available evidence on the consistency and strength of the association between smoking and dysmenorrhea.

Low heterogeneity and no significant publication bias were observed in this meta-analysis, indicating that the results of this study are relatively reliable and scientific. Nevertheless, sensitivity and subgroup analyses were performed to explore potential sources of heterogeneity. Therefore, the results suggested that the study conducted by Wood et al [20] was significantly responsible for a considerable amount of the heterogeneity. Subgroup analyses also reported an association between smoking and dysmenorrhea for all the defined stratifications.

Furthermore, results of this meta-analysis implied that the prevalence of dysmenorrhea among smokers was higher than that among non-smokers. This is in agreement with the assertion that smokers might experience more menstrual problems, such as prolonged periods, which have been related to dysmenorrhea [16]. In this regard, some studies have suggested that nicotine, a dominant substance in tobacco, could cause vasoconstriction, which may lead to myometrial contraction for the reason of resultant hypoxia [38]. Besides, the vasoconstriction leads to dysmenorrhea by decreasing the endometrial blood flow. Also, other studies have shown that nicotine could result in reduced endometrial blood flow, which is common in women with dysmenorrhea [39]. Referring to a prospective cohort study conducted in China, females who were exposed to high levels of second-hand smoke were at an increased risk of developing dysmenorrhea than those who were exposed to low levels of second-hand smoke, and that study attributed this phenomenon to the reason of decreasing endometrial blood flow, and resultant hypoxia [40]. Moreover, smoking may have a direct impact on the endocrine control of menstruation, as it is regularly related to some menstrual disorders, for example, prolonged periods [15], antiestrogenic extraovarian [41] and ovarian atrophy [42], which have been related to dysmenorrhea. Also, a previous study reported that quitting smoking could help women with dysmenorrhea by relieving symptoms [30]. Additionally, dysmenorrhea is thought to be caused by the release of prostaglandins in the menstrual fluid, which produces uterine contractions and pain. Therefore, although smoking is related to dysmenorrhea, more future research is needed to confirm this relationship.

Nonetheless, the findings of this meta-analysis provide a strong evidence that both former and current smoking may make women vulnerable to repeated and painful menstrual pain (dysmenorrhea) throughout their reproductive age. Thus, considering that smoking is a potentially modifiable factor associated with dysmenorrhea, it could serve as an important element in interventions aimed at reducing the prevalence of dysmenorrhea. Therefore, as most women are unaware about the association between smoking and dysmenorrhea, health education on this topic is an effective way to increase their awareness on this association, which may deter their smoking behaviour, and other intervention measures targeted at tobacco cessation might be useful in decreasing the prevalence of dysmenorrhea.

There are some limitations related to the findings of this study.

First, a significantly large proportion of the eligible studies used in this meta-analysis consisted of cross-sectional studies; hence causal connection of smoking to dysmenorrhea cannot be established in this meta-analysis.

Second, more high-quality studies are needed in the future, for this present meta-analysis used few high-quality studies, which may exaggerate the association between smoking and dysmenorrhea.

Third, moderate heterogeneity was observed when estimating the association between smoking and dysmenorrhea, which might affect the accuracy of the estimation. However, although study design and place of study might be sources of the heterogeneity, other factors that may also contribute to the heterogeneity were not explored due to inconsistent reporting of these in the eligible studies. For example, there was lack of uniformity in the diagnosis of dysmenorrhea (see Table 1), and there was no consensus on the definition of smoking, that is, the eligible studies divided females into former/current/never smokers, or smokers/non-smokers. Thus, the definition of dysmenorrhea relied on the subjective reporting of symptoms, which could underestimate or overestimate the prevalence of dysmenorrhea. In the same vein, subgroup analysis with respect to biological aspects could not be conducted due to limited data, for example, dose-response of smoking exposure was reported in only 2 eligible studies. Therefore, future studies need to report the foregoing factors consistently when examining the relationship between smoking and dysmenorrhea.

Lastly, factors which might be confounders for analysing the relationship between smoking and dysmenorrhea, such as age, race, perception of pain, BMI, family history of dysmenorrhea, age of menarche onset, and duration of dysmenorrhea were not considered when investigating the relationship between smoking and dysmenorrhea in the eligible studies. Therefore, future studies should consider these preceding factors when investigating the association between smoking and dysmenorrhea.

## 5. Conclusions

Dysmenorrhea has been an important public health problem and has a significant negative impact on the affected females. The evidence from this meta-analysis indicated a significant association between cigarette smoking (both current and former smoking) and dysmenorrhea. Therefore, the adverse effects of smoking provide further support for dysmenorrhea prevention plans and emphasize on the need for health intervention programs for females in the future.

## Supporting information

S1 Checklist PRISMA 2009 checklist. (DOC)

S1 Fig (TIF)

S2 Fig (TIF)

S3 Fig (TIF)

S4 Fig (TIF)

S5 Fig (TIF)

S6 Fig (TIF)

S1 Table The methodological quality assessment of case-control study(based on NOS). (DOCX)

S2 Table The methodological quality assessment of cohort study(based on NOS). (DOCX)

S3 Table The methodological quality assessment of cross-sectional study(Based on AHRQ). (DOCX)

## References

[1] Hailemeskel S, Demissie A, Assefa N. Primary dysmenorrhea magnitude, associated risk factors, and its effect on academic performance: evidence from female university students in Ethiopia. Int J Womens Health. 2016;8:489–96. 10.2147/IJWH.S112768 27695366 PMC5034908

[2] Subasinghe AK, Happo L, Jayasinghe YL, Garland SM, Gorelik A, Wark JD. Prevalence and severity of dysmenorrhoea, and management options reported by young Australian women. Aust Fam Physician. 2016;45(11):829–34. 27806454

[3] Habibi N, Huang MS, Gan WY, Zulida R, Safavi SM. Prevalence of Primary Dysmenorrhea and Factors Associated with Its Intensity Among Undergraduate Students: A Cross-Sectional Study. Pain Manag Nurs. 2015;16(6):855–61. 10.1016/j.pmn.2015.07.001 26328887

[4] Ortiz MI. Primary dysmenorrhea among Mexican university students: prevalence, impact and treatment. Eur J Obstet Gynecol Reprod Biol. 2010;152(1):73–7. 10.1016/j.ejogrb.2010.04.015 20478651

[5] French L. Dysmenorrhea. Am Fam Physician. 2005;71(2):285–91. 15686299

[6] Iacovides S, Avidon I, Baker FC. What we know about primary dysmenorrhea today: a critical review. Human reproduction update. 2015;21(6):762–78. 10.1093/humupd/dmv039 26346058

[7] De Sanctis V, Soliman AT, Elsedfy H, Soliman NA, Soliman R, El Kholy M. Dysmenorrhea in adolescents and young adults: a review in different country. Acta bio-medica: Atenei Parmensis. 2017;87(3):233–46. PMC10521891 28112688

[8] Midilli TS, Yasar E, Baysal E. Dysmenorrhea Characteristics of Female Students of Health School and Affecting Factors and Their Knowledge and Use of Complementary and Alternative Medicine Methods. Holist Nurs Pract. 2015;29(4):194–204. 10.1097/HNP.0000000000000091 26086463

[9] Harel Z. Dysmenorrhea in adolescents and young adults: etiology and management. J Pediatr Adolesc Gynecol. 2006;19(6):363–71. 10.1016/j.jpag.2006.09.001 17174824

[10] Ju H, Jones M, Mishra G. The prevalence and risk factors of dysmenorrhea. Epidemiol Rev. 2014;36:104–13. 10.1093/epirev/mxt009 24284871

[11] Fernandez-Martinez E, Onieva-Zafra MD, Parra-Fernandez ML. Lifestyle and prevalence of dysmenorrhea among Spanish female university students. PLoS One. 2018;13(8):e0201894 10.1371/journal.pone.0201894 30096156 PMC6086430

[12] Gagua T, Tkeshelashvili B, Gagua D. Primary dysmenorrhea: prevalence in adolescent population of Tbilisi, Georgia and risk factors. J Turk Ger Gynecol Assoc. 2012;13(3):162–8. 10.5152/jtgga.2012.21 24592031 PMC3939234

[13] Ozerdogan N, Sayiner D, Ayranci U, Unsal A, Giray S. Prevalence and predictors of dysmenorrhea among students at a university in Turkey. Int J Gynaecol Obstet. 2009;107(1):39–43. 10.1016/j.ijgo.2009.05.010 19539288

[14] Sundell G, Milsom I, Andersch B. Factors influencing the prevalence and severity of dysmenorrhoea in young women. Br J Obstet Gynaecol. 1990;97(7):588–94. 10.1111/j.1471-0528.1990.tb02545.x 2390501

[15] Parazzini F, Tozzi L, Mezzopane R, Luchini L, Marchini M, Fedele L. Cigarette smoking, alcohol consumption, and risk of primary dysmenorrhea. Epidemiology. 1994;5(4):469–72. 10.1097/00001648-199407000-00016 7918820

[16] Andersch B, Milsom I. An epidemiologic study of young women with dysmenorrhea. Am J Obstet Gynecol. 1982;144(6):655–60. 10.1016/0002-9378(82)90433-1 7137249

[17] Yamamoto K, Okazaki A, Sakamoto Y, Funatsu M. The relationship between premenstrual symptoms, menstrual pain, irregular menstrual cycles, and psychosocial stress among Japanese college students. J Physiol Anthropol. 2009;28(3):129–36. 10.2114/jpa2.28.129 19483374

[18] Unsal A, Ayranci U, Tozun M, Arslan G, Calik E. Prevalence of dysmenorrhea and its effect on quality of life among a group of female university students. Ups J Med Sci. 2010;115(2):138–45. 10.3109/03009730903457218 20074018 PMC2853792

[19] Seven M, Guvenc G, Akyuz A, Eski F. Evaluating dysmenorrhea in a sample of Turkish nursing students. Pain Manag Nurs. 2014;15(3):664–71. 10.1016/j.pmn.2013.07.006 24631318

[20] Wood C, Larsen L, Williams R. Social and psychological factors in relation to premenstrual tension and menstrual pain. Aust N Z J Obstet Gynaecol. 1979;19(2):111–5. 10.1111/j.1479-828x.1979.tb01367.x 292427

[21] Teperi J, Rimpela M. Menstrual pain, health and behaviour in girls. Soc Sci Med. 1989;29(2):163–9. 10.1016/0277-9536(89)90164-0 2787534

[22] Charlton A, While D. Smoking and menstrual problems in 16-year-olds. J R Soc Med. 1996;89(4):193–5. 8676315 10.1177/014107689608900405 PMC1295733

[23] Kritz-Silverstein D, Wingard DL, Garland FC. The association of behavior and lifestyle factors with menstrual symptoms. J Womens Health Gend Based Med. 1999;8(9):1185–93. 10.1089/jwh.1.1999.8.1185 10595332

[24] Strinic T, Bukovic D, Pavelic L, Fajdic J, Herman I, Stipic I, et al Anthropological and clinical characteristics in adolescent women with dysmenorrhea. Coll Antropol. 2003;27(2):707–11. 14746162

[25] Weissman AM, Hartz AJ, Hansen MD, Johnson SR. The natural history of primary dysmenorrhoea: a longitudinal study. Bjog. 2004;111(4):345–52. 10.1111/j.1471-0528.2004.00090.x 15008771

[26] Burnett MA, Antao V, Black A, Feldman K, Grenville A, Lea R, et al Prevalence of primary dysmenorrhea in Canada. J Obstet Gynaecol Can. 2005;27(8):765–70. 10.1016/s1701-2163(16)30728-9 16287008

[27] Patel V, Tanksale V, Sahasrabhojanee M, Gupte S, Nevrekar P. The burden and determinants of dysmenorrhoea: a population-based survey of 2262 women in Goa, India. Bjog. 2006;113(4):453–63. 10.1111/j.1471-0528.2006.00874.x 16489934

[28] Laszlo KD, Kopp MS. Effort-reward imbalance and overcommitment at work are associated with painful menstruation: results from the Hungarostudy Epidemiological Panel 2006. J Occup Environ Med. 2009;51(2):157–63. 10.1097/JOM.0b013e318197ca89 19209036

[29] Grandi G, Ferrari S, Xholli A, Cannoletta M, Palma F, Romani C, et al Prevalence of menstrual pain in young women: what is dysmenorrhea? J Pain Res. 2012;5:169–74. 10.2147/JPR.S30602 22792003 PMC3392715

[30] Ju H, Jones M, Mishra GD. Smoking and trajectories of dysmenorrhoea among young Australian women. Tob Control. 2016;25(2):195–202. 10.1136/tobaccocontrol-2014-051920 25403655

[31] Sahin S, Ozdemir K, Unsal A, Arslan R. Review of frequency of dysmenorrhea and some associated factors and evaluation of the relationship between dysmenorrhea and sleep quality in university students. Gynecol Obstet Invest. 2014;78(3):179–85. 10.1159/000363743 25171204

[32] Ibrahim NK, AlGhamdi MS, Al-Shaibani AN, AlAmri FA, Alharbi HA, Al-Jadani AK, et al Dysmenorrhea among female medical students in King Abdulaziz University: Prevalence, Predictors and outcome. Pak J Med Sci. 2015;31(6):1312–7. 10.12669/pjms.316.8752 26870088 PMC4744273

[33] Pejcic A, Jankovic S. Risk factors for dysmenorrhea among young adult female university students. Ann Ist Super Sanita. 2016;52(1):98–103. 10.4415/ANN_16_01_16 27033624

[34] Tomas-Rodriguez MI, Palazon-Bru A, Martinez-St John DR, Navarro-Cremades F, Toledo-Marhuenda JV, Gil-Guillen VF. Factors Associated with Increased Pain in Primary Dysmenorrhea: Analysis Using a Multivariate Ordered Logistic Regression Model. J Pediatr Adolesc Gynecol. 2017;30(2):199–202. 10.1016/j.jpag.2016.09.007 27693647

[35] Abu Helwa HA, Mitaeb AA, Al-Hamshri S, Sweileh WM. Prevalence of dysmenorrhea and predictors of its pain intensity among Palestinian female university students. BMC Womens Health. 2018;18(1):18 10.1186/s12905-018-0516-1 29334974 PMC5769430

[36] Orhan C, Celenay ST, Demirturk F, Ozgul S, Uzelpasaci E, Akbayrak T. Effects of menstrual pain on the academic performance and participation in sports and social activities in Turkish university students with primary dysmenorrhea: A case control study. J Obstet Gynaecol Res. 2018;44(11):2101–9. 10.1111/jog.13768 30043399

[37] Jenabi E, Khazaei SP, Veisani YP. The relationship between smoking and dysmenorrhea: A meta-analysis. Women Health. 2018:1–10. 10.1080/03630242.2018.1508541 30481133

[38] Speroff L, Fritz M. Clinical gynecologic endocrinology and infertility: lippincott Williams & wilkins 2011.

[39] Hornsby PP, Wilcox AJ, Weinberg CR. Cigarette smoking and disturbance of menstrual function. Epidemiology. 1998;9(2):193–8. 9504290

[40] Chen C, Cho SI, Damokosh AI, Chen D, Li G, Wang X, et al Prospective study of exposure to environmental tobacco smoke and dysmenorrhea. Environ Health Perspect. 2000;108(11):1019–22. 10.1289/ehp.001081019 11102290 PMC1240156

[41] Baron JA. Smoking and estrogen-related disease. Am J Epidemiol. 1984;119(1):9–22. 10.1093/oxfordjournals.aje.a113730 6362403

[42] Greenberg G, Thompson SG, Meade TW. Relation between cigarette smoking and use of hormonal replacement therapy for menopausal symptoms. J Epidemiol Community Health. 1987;41(1):26–9. 10.1136/jech.41.1.26 3668456 PMC1052572

