# Supplementary figures and images for: Association between cigarette smoking and the risk of dysmenorrhea: A meta-analysis of observational studies
Source: PLoS One. 2020 Apr 15;15(4):e0231201. doi: 10.1371/journal.pone.0231201 (PMC7159229; doi:10.1371/journal.pone.0231201)

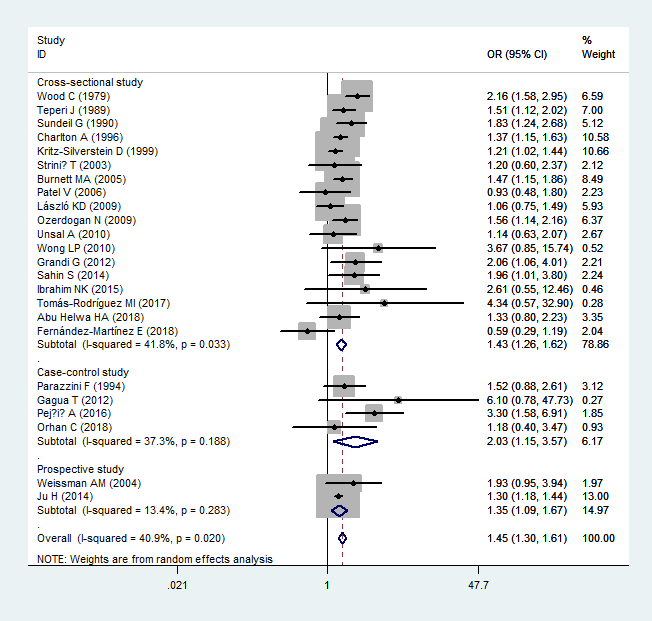

Supplement: S1 Fig — (TIF) [file pone.0231201.s002.tif]

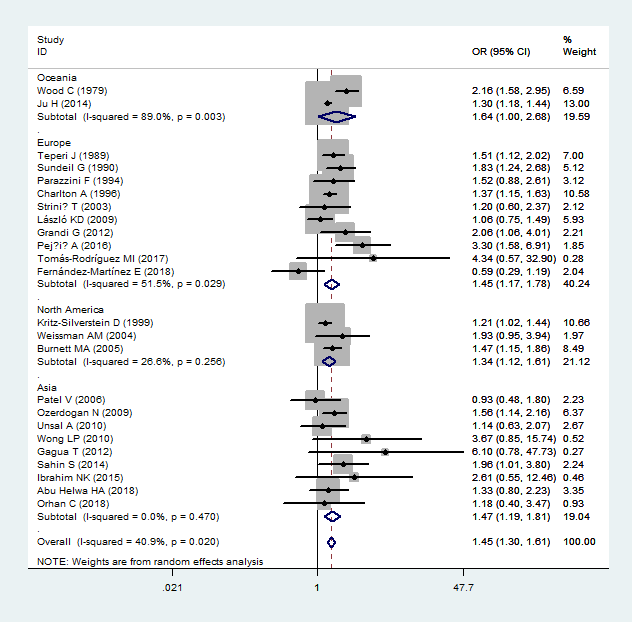

Supplement: S2 Fig — (TIF) [file pone.0231201.s003.tif]

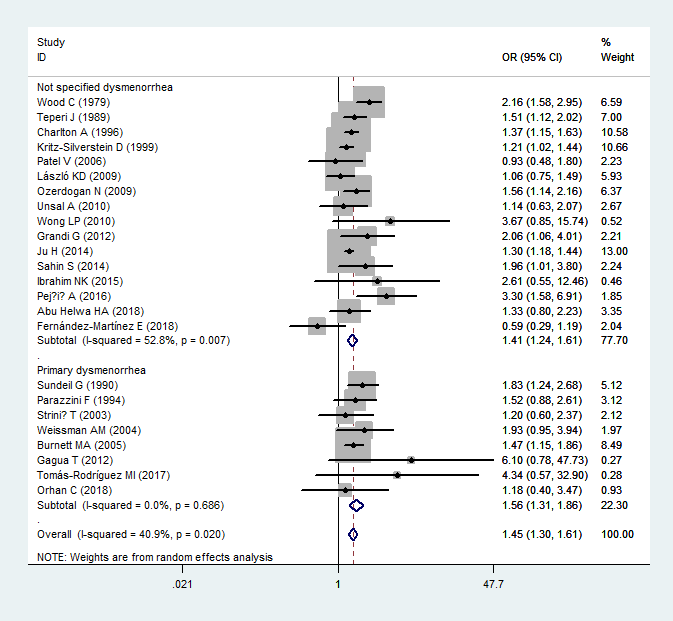

Supplement: S3 Fig — (TIF) [file pone.0231201.s004.tif]

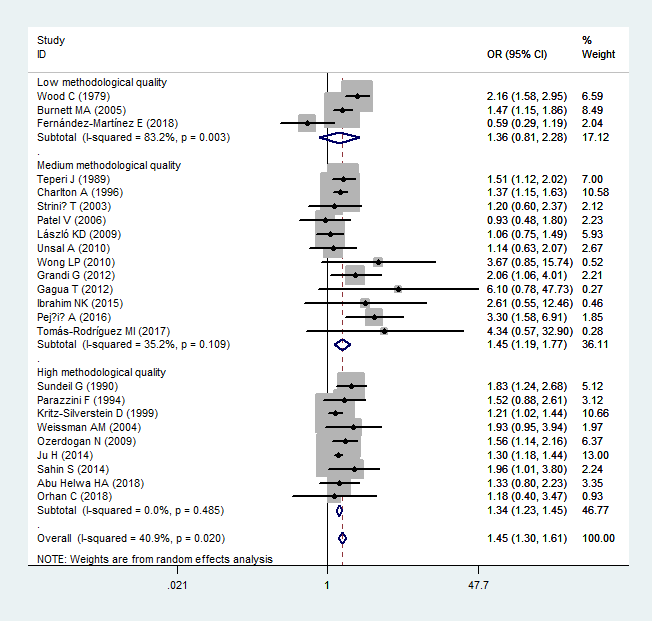

Supplement: S4 Fig — (TIF) [file pone.0231201.s005.tif]

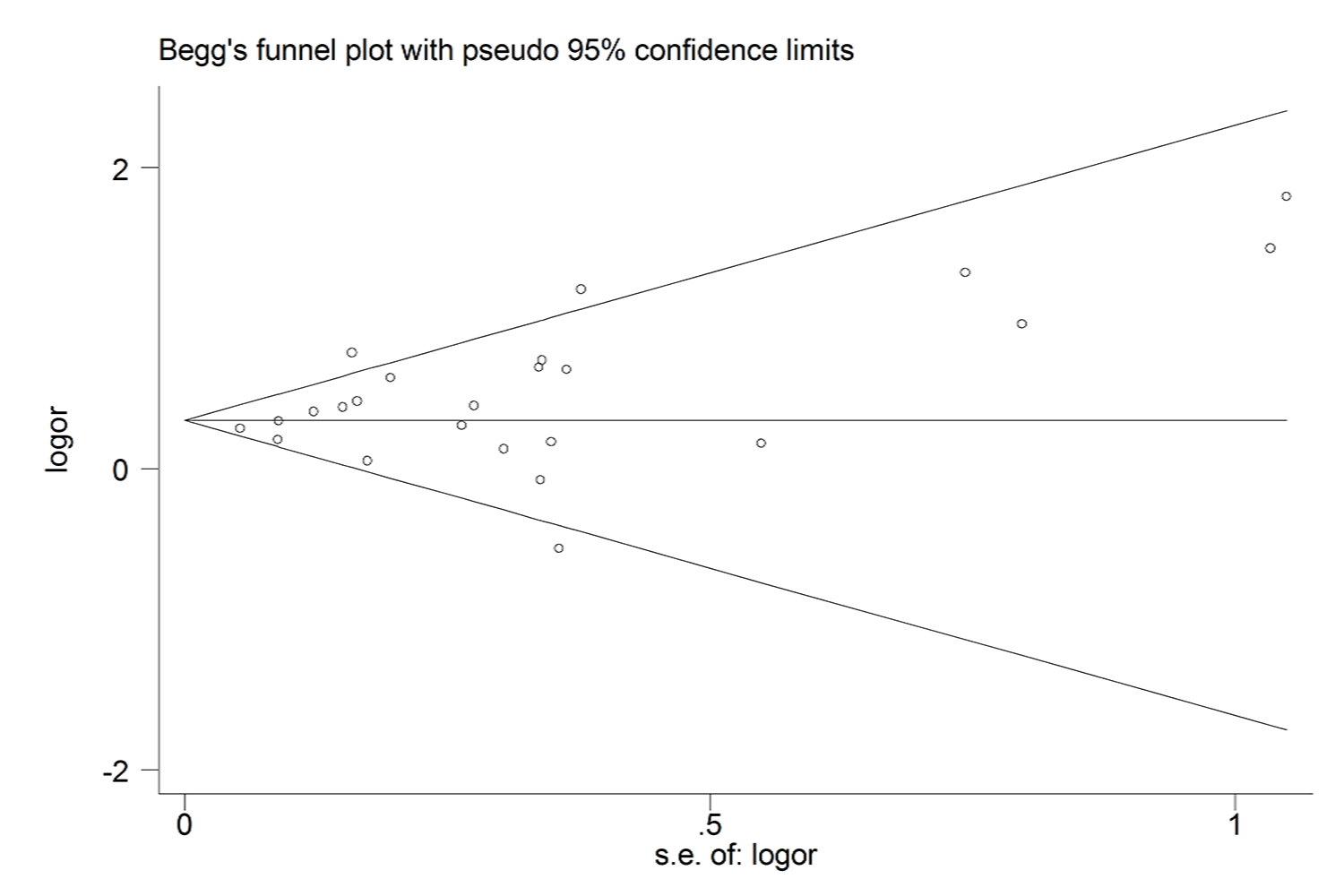

Supplement: S5 Fig — (TIF) [file pone.0231201.s006.tif]

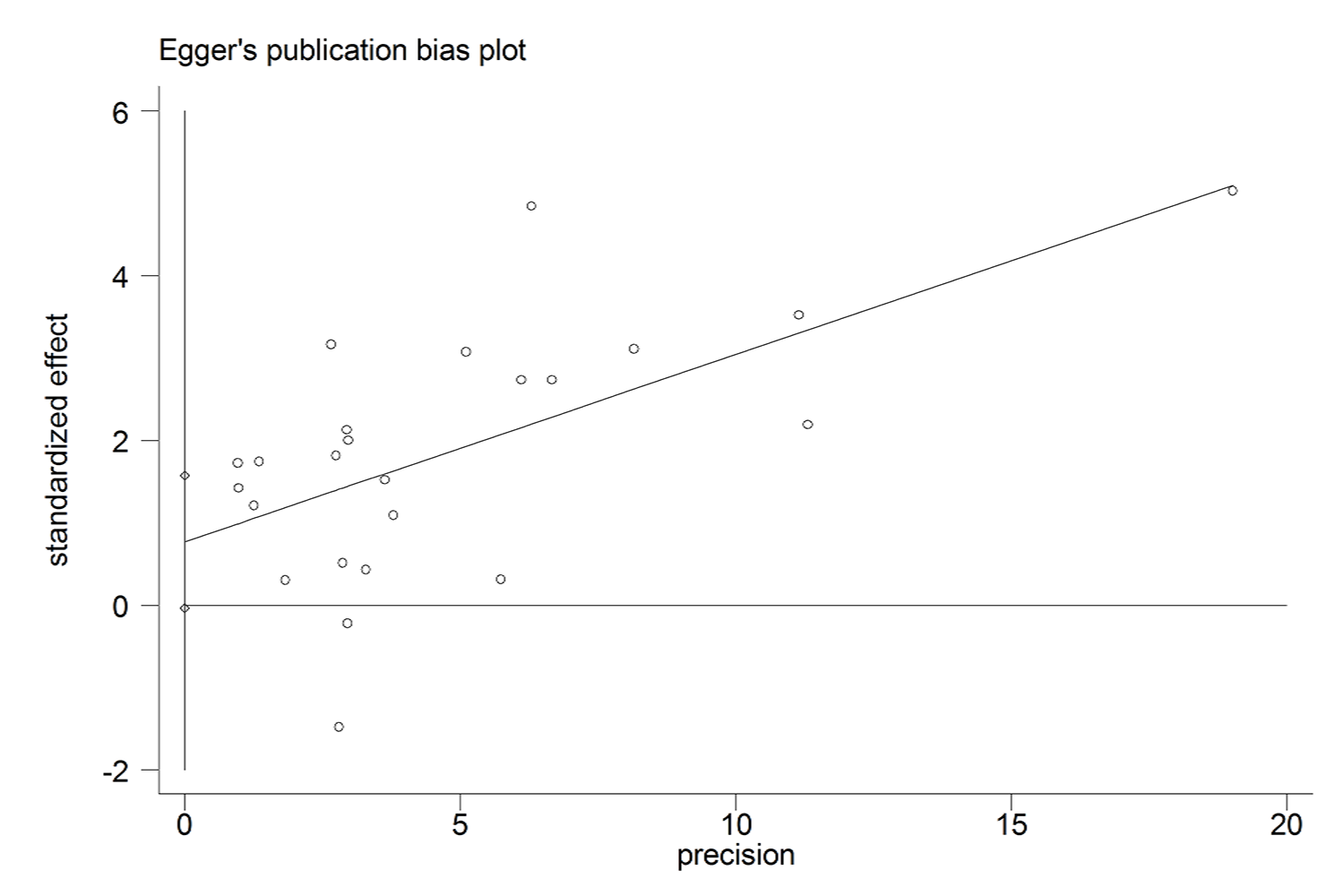

Supplement: S6 Fig — (TIF) [file pone.0231201.s007.tif]
