# Supplementary material for: Association between cigarette smoking and the risk of dysmenorrhea: A meta-analysis of observational studies
Source: PLoS One. 2020 Apr 15;15(4):e0231201. doi: 10.1371/journal.pone.0231201 (PMC7159229; doi:10.1371/journal.pone.0231201)
Supplement: S1 Table — (DOCX) [file pone.0231201.s008.docx]

**Supplement Table 1 The methodological quality assessment of case-control study(Based on NOS)**

| Study ID | Dysmenorrhea definition adequate | Representativeness of the cases | Selection of Controls | Definition of Controls | Comparability* | Ascertainment of smoking | Same method of ascertainment for cases and controls | Non-Response rate | Score | Quality |
| --- | --- | --- | --- | --- | --- | --- | --- | --- | --- | --- |
| Parazzini F(1994) | ✓ | ✓ | ✓ | ✓ | ✓ | • | ✓ | ✓ | 7 | high |
| Gagua T(2012) | • | ✓ | ✓ | ✓ | ✓ | • | ✓ | **×** | 5 | medium |
| Pejčić A(2016) | ✓ | ✓ | **×** | ✓ | ✓ | • | ✓ | **×** | 5 | medium |
| Orhan C(2018) | ✓ | ✓ | ✓ | ✓ | ✓ | • | ✓ | ✓ | 7 | high |

Note: •described the variable but cannot allocated a star ; *A maximum of 2 stars can be allotted in this category
