# Supplementary material for: Association between cigarette smoking and the risk of dysmenorrhea: A meta-analysis of observational studies
Source: PLoS One. 2020 Apr 15;15(4):e0231201. doi: 10.1371/journal.pone.0231201 (PMC7159229; doi:10.1371/journal.pone.0231201)
Supplement: S2 Table — (DOCX) [file pone.0231201.s009.docx]

**Supplement Table 2 The methodological quality assessment of cohort study(Based on NOS)**

| Study ID | Representativeness of the cohort | Selection of the non-exposed cohort | Ascertainment of smoking | dysmenorrhea was not present at start | Comparability* | Assessment of outcome | follow-up long enough for outcomes | Adequacy of follow up | Score | Quality |
| --- | --- | --- | --- | --- | --- | --- | --- | --- | --- | --- |
| Weissman(2004) | ✓ | ✓ | • | ✓ | ✓ | ✓ | ✓ | ✓ | 7 | high |
| Ju H(2014) | ✓ | ✓ | ✓ | ✓ | **×** | ✓ | ✓ | ✓ | 7 | high |

Note: •described the variable but cannot allocated a star ; *A maximum of 2 stars can be allotted in this category
