# Supplementary material for: Association between cigarette smoking and the risk of dysmenorrhea: A meta-analysis of observational studies
Source: PLoS One. 2020 Apr 15;15(4):e0231201. doi: 10.1371/journal.pone.0231201 (PMC7159229; doi:10.1371/journal.pone.0231201)
Supplement: S3 Table — (DOCX) [file pone.0231201.s010.docx]

**Supplement Table 3 The methodological quality assessment of cross-sectional study(Based on AHRQ)**

| Stydy ID | Item 1 | Item 2 | Item 3 | Item 4 | Item 5 | Item 6 | Item 7 | Item 8 | Item 9 | Item10 | Item 11 | Score | Quality |
| --- | --- | --- | --- | --- | --- | --- | --- | --- | --- | --- | --- | --- | --- |
| Wood C(1979) | ✓ | × | × | • | × | × | ✓ | × | ✓ | • | • | 3 | low |
| Teperi j(1989) | ✓ | × | ✓ | ✓ | • | × | × | × | × | ✓ | • | 4 | medium |
| Sundell G(1990) | ✓ | ✓ | ✓ | ✓ | • | • | ✓ | • | ✓ | ✓ | ✓ | 8 | high |
| Charlton A(1996) | ✓ | × | × | • | • | × | × | × | ✓ | ✓ | ✓ | 4 | medium |
| Kritz-Silverstein D(1999) | ✓ | ✓ | ✓ | ✓ | • | ✓ | ✓ | ✓ | ✓ | ✓ | • | 9 | high |
| Strinić T(2003) | ✓ | ✓ | ✓ | • | • | ✓ | × | × | × | × | • | 4 | medium |
| Burnett MA(2005) | ✓ | ✓ | ✓ | • | • | × | × | • | • | × | • | 3 | low |
| Patel V(2006) | ✓ | × | ✓ | • | • | ✓ | ✓ | ✓ | ✓ | ✓ | • | 7 | medium |
| László KD(2009) | ✓ | ✓ | ✓ | ✓ | • | × | × | • | ✓ | ✓ | • | 6 | medium |
| Ozerdogan N(2009) | ✓ | ✓ | ✓ | × | • | ✓ | ✓ | ✓ | ✓ | ✓ | • | 8 | high |
| Unsal A(2010) | ✓ | × | ✓ | • | • | ✓ | ✓ | • | × | ✓ | × | 5 | medium |
| Wong LP(2010) | ✓ | × | × | × | • | × | ✓ | ✓ | ✓ | ✓ | × | 5 | medium |
| Grandi G(2012) | ✓ | ✓ | ✓ | • | • | × | × | × | ✓ | ✓ | • | 5 | medium |
| Sahin S(2014) | ✓ | ✓ | ✓ | • | × | ✓ | ✓ | ✓ | ✓ | ✓ | • | 8 | high |
| Ibrahim NK(2015) | ✓ | ✓ | ✓ | • | • | ✓ | × | × | × | × | • | 4 | medium |
| Tomás-Rodríguez MI(2017) | ✓ | × | × | • | • | ✓ | ✓ | × | • | ✓ | • | 4 | medium |
| Abu Helwa HA(2018) | ✓ | ✓ | ✓ | • | • | ✓ | ✓ | ✓ | ✓ | ✓ | • | 8 | high |
| Fernández-Martínez E(2018) | ✓ | × | ✓ | • | • | • | × | × | • | ✓ | × | 3 | low |

Note:✓: yes; ×:no; •:unclear
